# Supplementary material for: MetaRibo-Seq measures translation in microbiomes
Source: Nat Commun. 2020 Jun 29;11:3268. doi: 10.1038/s41467-020-17081-z (PMC7324362; doi:10.1038/s41467-020-17081-z)
Supplement: Supplementary file 10 — Supplementary Data 7 [file 41467_2020_17081_MOESM10_ESM.zip › File2/Confidence_High_Taxonomy/409471_out.krona.html]

Javascript must be enabled to view this page.

members
magnitude
magnitudeUnassigned
count
unassigned
taxon
rank

409471\_out

42

2
superkingdom
42

42
phylum
1239

42
class
186801

order
42
186802

42
family
541000

1898205
42
species

SRS011134\_contig\_number\_38209SRS012849\_contig\_number\_2609SRS015663\_contig\_number\_15008SRS015782\_contig\_number\_16135SRS016203\_contig\_number\_8794SRS017701\_contig\_number\_8929SRS017916\_contig\_number\_1758SRS018541\_contig\_number\_12284SRS020394\_contig\_number\_11214SRS021948\_contig\_number\_contig-100\_992.186438SRS022609\_contig\_number\_24714SRS024132\_contig\_number\_47088SRS045004\_contig\_number\_contig-100\_1425.175915SRS045645\_contig\_number\_8064SRS047044\_contig\_number\_contig-100\_933.81656SRS048164\_contig\_number\_14843SRS049995\_contig\_number\_15331SRS051031\_contig\_number\_16979SRS052697\_contig\_number\_18627SRS054059\_contig\_number\_491SRS058070\_contig\_number\_22514SRS063518\_contig\_number\_contig-100\_191.232583SRS064757\_contig\_number\_3017SRS075341\_contig\_number\_19246SRS075773\_contig\_number\_contig-100\_527.203263SRS077392\_contig\_number\_33438SRS078176\_contig\_number\_contig-100\_794.126161SRS078419\_contig\_number\_30444SRS101376\_contig\_number\_28434SRS1041092\_contig\_number\_12966SRS1041136\_contig\_number\_4423SRS1041141\_contig\_number\_10085SRS1041145\_contig\_number\_contig-100\_577.178917SRS104400\_contig\_number\_3608SRS1055083\_contig\_number\_7028SRS143895\_contig\_number\_contig-100\_813.248074SRS144537\_contig\_number\_1120SRS147271\_contig\_number\_9132SRS147346\_contig\_number\_65693SRS148196\_contig\_number\_contig-100\_67.192684SRS150029\_contig\_number\_16133SRS971276\_contig\_number\_3091
